# The Optimisation of Cooking Parameters for Spirt Whiskey Production from Native Irish Wheat: A Response Surface Method Approach

**DOI:** 10.3390/foods11091199

**Published:** 2022-04-20

**Authors:** Sinead Morris, John L. Byrne, Ben Murphy, Stephen J. Whelan, John P. Carroll, David Ryan

**Affiliations:** EnviroCORE, Department of Science and Health, Institute of Technology Carlow, R93 V960 Carlow, Ireland; john.byrne@itcarlow.ie (J.L.B.); c00242562@itcarlow.ie (B.M.); stephen.whelan@itcarlow.ie (S.J.W.); john.carroll@itcarlow.ie (J.P.C.); david.ryan@itcarlow.ie (D.R.)

**Keywords:** alcohol yields, response surface methods, process optimisation, wheat

## Abstract

Barley and maize have dominated the Irish whiskey sector, but in recent years, alternative grains have started to gain traction. Ireland has a high average wheat yield, producing grain that is high in starch but low in protein, offering the potential for use in distillation. To successfully utilise Irish-grown wheat in distillation, cultivars that are suitable to the Irish climate and give high yields of alcohol need to be identified. This necessitates the development of a rapid screening test for grain alcohol yield. This study examined the optimal temperature, time period, α-amylase dose rate, and calcium concentration to be used in the cooking of wheat grain to maximise alcohol yields. It was determined that lower cooking temperatures are more successful in achieving higher alcohol yields, and it was confirmed that temperature is a key variable in the cooking process. By optimising all parameters, alcohol yields of 458 LA/tonne were obtained, demonstrating that the optimum parameters can be successfully used for both hard and soft endoderm wheat produced in Ireland as well as for different varieties. This indicates potential for producing higher alcohol yields using Irish-grown wheat in Irish distilleries.

## 1. Introduction

Despite the fact that the Irish tillage sector produces 0.6 million tonnes of wheat grain per annum [1,2], the use of Irish grown wheat in the domestic distilling sector is minimal, with barley and maize still dominant [1]. While barley is the most common cereal grown in Ireland, maize grain is imported, thus undermining the provenance of Irish whiskey. By contrast, Ireland has a high average wheat yield, producing grain that is high in starch and low in protein, making Irish wheat ideal for producing significant alcohol yields (AY) [2]. In order to determine the viability of utilising Irish wheat in distilleries, research is required to determine Irish-grown varieties’ ability to achieve high AY. 

There are four potential factors involved in cooking grain: timeframe, temperature, α-amylase dose rate, and calcium (Ca) ions concentration required to stabilise the enzymes. Typically, high-temperature grain cooking is used to gelatinise starch, with enzymes such as α-amylase aiding liquefaction [3]. There has been little research into optimising cooking parameters of wheat for spirit alcohol in an Irish context due to the nature of the processes employed alongside perceived processability issues, such as viscosity and excessive foaming. In most Irish distilleries, grains are cooked at temperatures ranging from 92 °C to 105 °C, with the addition of commercial enzymes and calcium. However, research comparing high-temperature cooks (142 °C) with low-temperature cooks (85 °C) [3,4,5,6] have indicated that lower temperatures result in higher AY for both hard and soft wheat.

The key issues that must be addressed are whether lower cooking temperatures may be used in alcohol production utilising Irish wheat and thus determining any processability that may be observed during this process. An optimal standard procedure for the cooking technique is required to ensure a consistent process for testing small batches of wheat for AY potential. This research focuses on the optimisation of the cooking stage in the production of alcohol from Irish-grown wheat grain to obtain the greatest AY in a system that mimics Irish distilleries. The research concentrates on hard wheat (cv. Costello), which is the main wheat type cultivated in Ireland because it is well-adapted to both cli-mate and environmental conditions. However, hard wheat contains more protein than soft wheat, which may be one of the reasons distillers avoid using it due to concerns about increased viscosity and foaming during fermentation [3]. Hardness has also been connected to processing concerns, with the starch being less accessible and causing handling problems [7,8].

The work utilised a laboratory-based approach, which has been developed by the authors and mirrors Irish industry practices for optimising cooking parameters. This was carried out through experimental design emphasising response surface methods. The objective was to identify the best parameters for cooking wheat samples while considering the roles that temperature, time, calcium, and α-amylase play in the process and to deter-mine their optimum values. The main goals were both to establish the optimum cooking process for the liquefaction of starch to fermentable sugars in small batches of wheat that meet Irish industrial standards and to determine whether the optimum parameters are suitable for different wheat varieties and endosperm types.

## 2. Materials and Methods

### 2.1. Cereal Grain and Composition

Hard wheat grain (cv. Costello), supplied by Goldcrop Ireland, was grown and harvested in Ireland during 2019. Samples were stored in cool (4 °C), dark conditions until required for use. Soft wheat samples 1, 2, and 3 were supplied by Irish distilling industry professionals. Malted barley (cv. Laureate) was utilised during mashing. Before use, the grains were also kept in cool, dark conditions. Costello had a moisture content of 14%, and the soft wheat samples 1, 2, and 3 had a moisture content of 13.5%, 12%, and 13.75%, respectively.

### 2.2. Alcohol Yield Analysis

The method was based on that of Agu et al., 2006 [9], which stimulates the production process conditions in a “typical” Scotch whiskey grain distillery but modified for a “typical” Irish grain distillery. The main differences relate to the use of enzymes and process temperature and times. Wheat flour (30 g) was obtained by milling the grains in a Buhler Miag disc mill (setting 0.2 mm) (Buhler Group, Dublin, Ireland). This was transferred into a mashing beaker and slurried with water (86 mL preheated to 40 °C) containing a high-temperature-tolerant α-amylase (0–66 U/g of flour) (source: Bacillus licheniformis, working temperature range 45–95 °C, working pH range 3.8–8.5. Trade name: Termamyl (novozymes), Supplier: Sigma Aldrich, Dublin, Ireland) and 0–200 mg/L Ca ions in the form of calcium chloride dihydrate (CaCl_2_·2H_2_O; ranged from 0–733.66 mg/L; PanReac Appliedchem ITW reagents, Dublin, Ireland). The contents were gradually heated to either 72 °C, 82 °C, or 92 °C (temperature rise 2 °C/min) in a water bath and cooked for 60–150 min. The cooked slurry was then cooled to 66 °C and given a second treatment of α-amylase (38 U/g of flour) (Sigma Aldrich, Dublin, Ireland) and amyloglucosidase (0.22 U/g of flour) (Sigma Aldrich, Dublin, Ireland). This was mashed for 75 min, with a malt inclusion rate of 5% using high diastatic power-distilling malted barley (cv. Laureate, Miag setting 0.2 mm). After cooling to 22 °C, the mash was pitched with distiller’s yeast (Pinnacle ‘M’ type, (AB Mauri), WHC labs, Wicklow, Ireland) at a pitching rate of 0.4% (*w*/*w*) and adjusted to 250 g with water. The mash was then fermented for 72 h at 30 °C with the addition of β-Glucanase (1.5 U/g of flour) (Sigma Aldrich, Dublin, Ireland). The alcohol yield was determined from the alcohol strength of the distillate, which was measured using an Anton Paar 5000 density meter (Anton Paar, Dublin, Ireland). The AY is quoted as litres of alcohol per tonne (LA/tonne) on a dry weight basis (dwb).

### 2.3. Comparison of Cooking Temperatures

**Figure 1:**
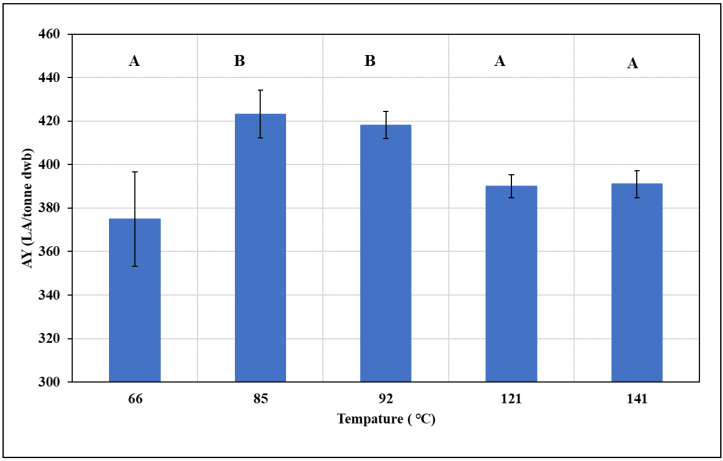
Comparison of AY achieved during the initial comparison of cooking temperature on hard wheat. All results presented mean ± standard deviation. Statistical analysis was employed, and samples with the same letter indicated no significant difference.

The effect of high and low cooking temperatures on AY was investigated. The protocol remains similar to that described in Section 2.2 for low cooking temperatures. During cooking, α-amylase was set at a dose rate of 66 U/g and calcium at 150 mg/L, and the timeframe was 150 min. Temperatures varied from 66–92 °C (temperature rise 2 °C/min) (Figure 1). The cooked slurry was then treated as described in Section 2.2.

Wheat flour (30 g) was obtained by milling the grains in a Buhler Miag disc mill (setting 0.2 mm). This was transferred into a mashing beaker and slurred with water (86 mL preheated to 40 °C). α-Amylase (66 U/g) and Ca (150 mg/L) were added before heating to 80 °C in a water bath. Samples were heated further to either 121 °C or 142 °C for 30 min. Post cooking, the cooked slurry was treated using the mashing conditions described in Section 2.2. All samples were run in triplicate. One-way ANOVA with Tukey post hoc test was conducted to test the null hypothesis (Ho) to ensure that there is no difference in AY when different cooking temperatures are employed.

### 2.4. Experimental Procedure: Response Surface Methods

A response surface methodology study was conducted to determine the relative contributions of four predictor factors: (A) time (minutes), (B) temperature (°C), (C) α-amylase (U/g), and (D) calcium (mg/L) to final alcohol yield (AY). Values of each predictor factor were based on previous literature [7,10,11], discussion with industry-based experts, and professional judgment. The cooking step ranged in temperature from 72–92 °C for 60–150 min, with α-amylase ranging from 0–66 U/g and calcium (in the form of CaCl_2_·H_2_O) varying from 0–200 mg/L. A central composite design (CCD), with two-level full factorial with added centre and axial point, was used due to it being a widely used statistical method based on the multivariate nonlinear model [12]. It encompassed a face-centred cube with triple-replicated factorial (blocks on replicates were applied), and the centre point was constructed using the software package Minitab^®^ V.20.01. Three levels of each predictor were incorporated into the design. Table 1 shows the value range for each component and the combination of these levels used in the face-centred cube. The response variable used to measure the optimum process was AY (LA/tonne dwb). A multiple regression analysis of the data was carried out by surface response methodology and the second-order polynomial equation that defines predicted responses (Yi) in terms of the independent variables:

(1) $$\begin{array}{cc} Y_{i}=b0_{i}+b1_{i}A & +b2_{i}B+b3_{i}C+b4_{i}D+b11_{i}\mathrm{AA}+b22_{i}\mathrm{BB}+b33_{i}\mathrm{CC}+b44_{i+}\mathrm{DD}+b12_{i}\mathrm{AB}+\;b13_{i}\mathrm{AC}+b14_{i}\mathrm{AD}\\ & +\;b23_{i}\mathrm{BC}+b24_{i}\mathrm{BD}+b34_{i}\mathrm{CD} \end{array}$$

where Y_i_ = predicted response; b0_i_ is intercept term; b1_i_, b2_i_, b3_i_, and b4_i_ are linear coefficients; b11_i_, b22_i_, b44_i_, and b44_i_ are squared coefficients; and b12_i_, b13_i_, b14_i_, b23_i_, b24_i_, and b34_i_ are interaction coefficients. A combination of factors represents an interaction between the individual factors in the respective term. These responses are a function of the level of factors. The response surface graphs indicate the effect of variables individually and in combination and determine their optimum levels. 

### 2.5. The Regression Equation and Predictive Analysis

Minitab was used to calculate regression equations (RE) from the RSM data output. The RE was analysed according to Section 2.2. The accuracy of the RE was determined by varying the amount of grain and grain size, as indicated in Table 2. After determining AY, mean percentage errors (MPE) were calculated, with a negative percentage error indicating an underperforming model and a positive percentage error suggesting a model that was overperforming. The MPE was calculated according to the equation (Equation (2)):

(2) $$\mathrm{MPE}=\frac{100\%}{n}\sum_{i=1}^{n}\frac{AY_{exp}-AY_{cal}}{AY_{exp}}$$

where AY_exp_ is the experimental value obtain during experiments, and AY_cal_ was obtained from the regression equation.

### 2.6. Soft versus Hard Wheat

Post optimisation, additional wheat samples were studied to determine if different varieties and types of endosperms have an impact on the AY and to confirm whether the chosen optimal cooking temperature is indeed suitable for both hard and soft wheat types. Three soft wheat varieties were examined. All samples were cooked using optimum conditions, following the method described in Section 2.2. A cooking temperature of 78 °C was set for 123 min, using 66 U/g α-amylase and 141 mg/L Ca. An additional study was carried out using optimal parameters but altering temperatures. Temperature profiles ranged from low-temperature (72 °C, 78 °C, 82 °C, 85 °C, and 92 °C) to high-temperature (121 °C and 142 °C). One-way ANOVA with Tukey post hoc test was carried out to test the null hypothesis (Ho) that no differences in the mean AY would be obtained. 

## 3. Results

### 3.1. Comparison of Cooking Temperatures 

Multiple variables must be evaluated to begin optimising the cooking process in a way that reflects industrial norms. To initiate the process of liquefaction of starch to fermentable sugar, α-amylase and calcium are needed, but wheat starch must first reach its gelatinisation temperature (51–60 °C) [13]. Some research shows that high-temperature cooking (142 °C) [4] will provide the highest AY, and others advocate cooking at a lower temperature, 85 °C [5,9], while personal communications with the Irish industry specialists suggest 92 °C. Grains such as maize require greater cooking temperatures to achieve higher AY [9], whereas wheat can be processed at lower temperatures, and yields can be increased [4,11]. Green et al. [4] investigated this and stated that a cooking temperature of 85 °C produces more alcohol than using higher temperatures. Because of previous studies that examined AY at this temperature [4,9,11], it was decided to test these temperatures (85 °C and 142 °C) along with the current industry standard of 92 °C. Furthermore, because mashing takes place at 66 °C, this was considered a suitable temperature to study for comparisons to 121 °C, which is the temperature used for autoclaving (Figure 1).

AY ranged from 375 to 423 LA/tonne dwb (litres of alcohol produced by a tonne of grain on a dry weight basis) during temperature cooking profiles (Figure 1). Initially, AY appeared to have a low mean variation. However, the null hypothesis that all mean AYs are equal is rejected (*p*-value 0.003). There is a substantial variation in the mean AY between the temperatures. When samples were cooked at 85 °C and 92 °C (Figure 1), the Tukey post hoc test demonstrated no variations in AY, with mean AY of 423 and 418 LA/tonne dwb, respectively. During this trial, these were the highest yields. The mean AY at 121 °C and 142 °C was 390 and 391 LA/tonne dwb, respectively, while the mean AY at 66 °C was 375 LA/tonne dwb (Figure 1), with no discernible differences between these temperature ranges. At 85 °C, the AY of 423.07 LA/tonne is comparable to yields obtained by other researchers for various types of feedstocks [3,9]. When wheat (cv. Viscount) was cooked at low temperatures, Green et al. [4] reported AY of 454–459 LA/tonne, while Agu et al. [11] reported AY of 467–482 LA/tonne with low-nitrogen wheat (1.24–1.53%) and 413–435 LA/tonne with higher-nitrogen wheat (1.83–2.14%). At 142 °C, AY of 391.97 LA/tonne dwb was achieved in this study. This was much lower than what had previously been reported in the literature. It is worth noting that these studies primarily focused on soft wheat, thus comparing AY across different types may be pointless in this case, especially when processing circumstances vary slightly. When hard wheat (cv. Option) was cooked at 142 °C, one study reported an AY of 439–462 LA/tonne, with better yields recorded in samples with a low-nitrogen rate applied to them during field studies [10]. The AY achieved in this study was significantly lower, implying that additional parameters employed during cooking, such as enzymes and calcium loading rates, must be optimised. This emphasises the importance of examining each independent variable in cooking to optimise each parameter. Temperature is important, but the influence of time, α-amylase, and calcium ions must also be considered.

### 3.2. RSM Analysis of Cooking Process

**Figure 2:**
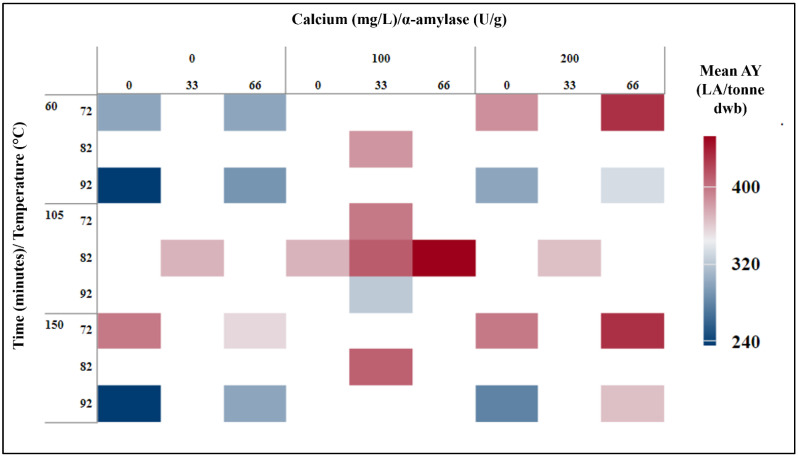
Heat map displaying experimental runs used during RSM, with mean AY obtained.

The RSM method uses design-of-experiment techniques to determine the optimal level of each parameter in relation to the dependent variable. This method allows for the detection of important variables in the process as well as examining how each variable interacts to reach the common aim of higher AY. The four independent variables were investigated in a variety of experimental setups (Figure 2). In order to optimise each variable, focus was placed on the amount of α-amylase required as well as calcium and temperature profiles in order to maximize AY while also considering the interaction between variables and their impact on AY. Depending on the experimental parameters, AY ranged from 240 to 450 LA/tonne dwb (Figure 2). Different combinations of the independent variable (Ca, time, temperature, and α-amylase) resulted in varying AYs. It was evident that some combinations produced minimal alcohol when compared to other combinations. For example, at 92 °C, the lowest yields are observed, while larger yields are observed at 72 °C and 82 °C. As a result, in order to determine the most appropriate parameters for use in optimising AY, a more detailed statistical assessment of the findings was required.

#### 3.2.1. Statistical Analysis 

The aim of statistical analysis is to determine which independent variables have an impact on the final AY, alongside helping in determining if there is any interaction between independent variables and their impact on AY. The overall model’s analysis of variance yields a *p*-value of <0.001, indicating that the null hypothesis of no relationship between the independent and dependent variables may be rejected (Table 3). This means that each independent variable affects the AY obtained throughout the model. Furthermore, the *p*-value for the blocks on replicates is 0.139, showing that there are no significant changes in blocks, indicating true replicates (Table 3). The independent factors explain the variance in the AY obtained, according to the adjusted R-square value of 89.98%, showing that the model has good practical importance (Table 4).

In terms of the linear independent variables, there is a substantial disparity in AY yields (*p*-value 1 × 10^−4^ (Table 3). According to the F-value (2.61), time is the least important variable in the system, indicating that there is no substantial change in AY over time. This showed that AY differed only slightly depending on the timeframe (90–150 min), with mean AY ranging from 328 to 394 LA/tonne dwb. Temperature appears to be the most important variable in the linear and overall system (F-value 34.46, contribution 27.27%) (Table 3). The *p*-value for temperature is 7 × 10^−6^, showing that there is a substantial difference in AY between the temperature profile investigations (Table 3). There is no significant difference in AY between 72 °C and 82 °C (*p*-value 0.429) according to one-way ANOVA with Tukey post hoc test; however, there is a difference between both temperature profiles and 92 °C (*p*-value 0.028). The mean ± standard deviation AY varied by temperature profile, with the lowest AY of 295 ± 42.6 in 92 °C, the highest AY of 400.7 ± 38.4 in 82 °C, and 72 °C yielding 376.8 ± 49.2. The standard deviation is rather high; however, these figures are based on temperature values as a singular independent variable. During the RSM experiments, the linear term of α-amylase yielded a *p*-value of 0.017, demonstrating variability in AY. The amount of α-amylase added during the process, as expected, had a considerable impact on the amount of AY obtained. This is due to α-amylase being essential for the conversion of starch to fermentable sugars. When 33 and 66 U/g α-amylase were utilised, there was no significant change in AY. However, when no α-amylase was used, there was a drop in AY of over 40 LA/tonne. In addition, grain swelled and remained gelatinised until mashing, when more enzymes (second dose of α-amylase and AMG) and malt were introduced, an indication of observed large-scale processability difficulties. 

**Figure 3:**
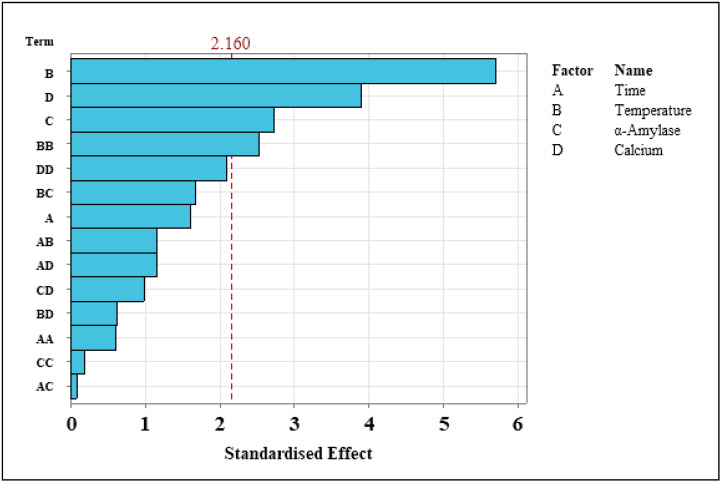
Pareto charts examining the standardised effects of the independent variable on the dependent variable. All independent variables above the 2.160 line indicated significant differences, meaning they have an important impact on the AY achieved.

Calcium (Ca) is the second most important independent variable in terms of linear terms (F-value 9.21, contribution 12.75%) (Table 3). The AY yield observed when varied quantities of Ca are used shows that Ca has a significant impact (*p*-value 0.002) (Table 3). Alcohol yields increased by over 50 LA/tonne when 100 and 200 mg/L of Ca were employed. Furthermore, it is known that Ca is essential for the stabilisation of α-amylase and maintaining the correct pH and that having the correct Ca concentration will have a significant impact on the potential AY [13,14]. Overall, the linear terms exhibited significant differences, bar time when they are studied as the singular independent variable affecting the dependent variable, AY. Figure 3 depicts the standardised impacts of both the independent and dependent factors in response to the dependent variable. The terms are listed in ascending order of importance. Temperature clearly has the greatest impact on AY, as previously stated, followed by both calcium and amylase having an impact on the yields achieved. Temperature squared is the final factor with a considerable impact, showing that temperature is the controlling phase in the process. This implies that, while the other variables are essential, the effect of temperature on AY is critical, accounting for a total contributing factor of 38.2% (Table 3).

The quadratic terms consider the squared power of each independent variable. There are significant effects here (*p*-value < 0.001) but not across all independent variables (Table 3). This suggests that some of the independent variables, such as α-amylase, are linear, and as a result, the AY should rise as the concentration rises until all starch is converted into fermentable sugars. There is no significant differentiation between the linear and quadratic values of time. In terms of the power squared, both temperature (*p*-value 0.025) and Ca (*p*-value 0.047, Table 3) are demonstrated to have a substantial impact on AY. This means that both independent variables will have a maximum value at which the maximum AY will be achieved, which will be discussed further in Section 3.2.2. The two-way interaction produces some interesting results (Table 3). It implies that there is not a substantial difference between any of the independent variable combinations and their impact on AY. In this case, the analysis of variance rejects the null hypothesis that each independent variable would interact with another to create an effect on AY, implying that each independent variable impacts AY by itself without the assistance of other factors. The variance inflation factor (VIF), a multicollinearity test, demonstrated that the dependent variables are unrelated (Table 3). This test measures how much the variance of an independent variable is influenced by its interaction with the other independent variables. All results are for the two-way interaction of variables are one (Table 3), indicating that they are not correlated, and therefore, variables studies are not influenced by other variables and their interactions. 

There is no association between variables when the value is less than one. This is interesting because it was predicted that α-amylase and Ca interact to have a combined effect on AY, as the literature states that adequate Ca ions are required to stabilise α-amylase and that α-amylase is rapidly denatured without Ca ions [14,15]. Further research will be required to ascertain whether there is interaction at play.

#### 3.2.2. Functional Analysis

**Figure 4:**
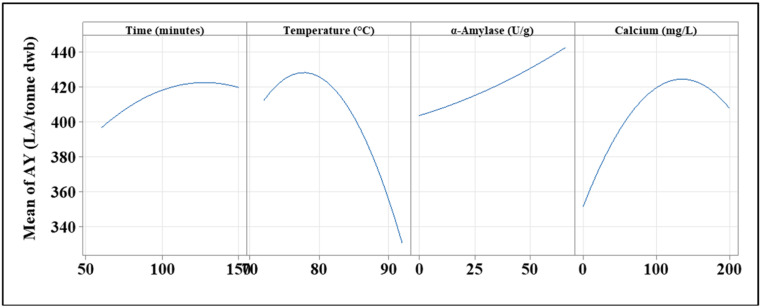
Main effect plot for the independent variables used within the study. Each variable is plotted against the mean AY achieved during the RSM study.

**Figure 5:**
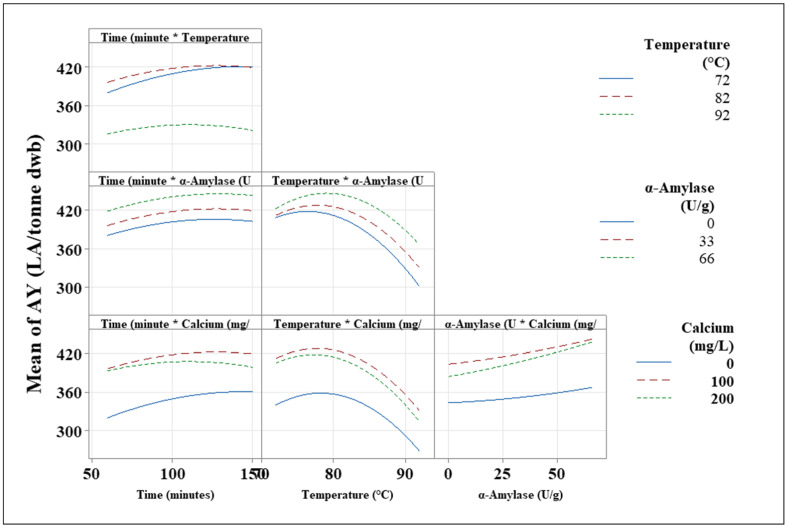
Interaction plots displaying the effect that combined independent variables have on the mean AY during the RSM model.

To determine how each parameter, both independently and in combination with other factors, impacts AY, the functional relationship between each variable’s impact on AY was investigated. This was accomplished using main effects plots (Figure 4), interaction plots (Figure 5), and the regression equation (Equation (3)) generated during the RSM data analysis. Each variable’s impact on AY is depicted as a single factor in the main effect plot (Figure 4). Examining the graphs, it is evident that the duration of the cooking process has a limited impact on AY. This term has a small curve, indicating that the AY is at its optimum when the time frame is longer than 100 min (Figure 4). Positive polynomial graphs are evident in both temperature and calcium (Figure 4). This means that when each of these factors is set to its maximum value, the maximum AY can be achieved. It was noted that at temperatures ranging from 76 °C to 79 °C, greater AY is achieved, with approximately 425 to 430 LA/tonne being produced. Furthermore, Ca has been shown to have a concentration that is optimal for AY. Ca levels should be between 130 and 145 mg/L for optimal AY results. α-amylase differs, displaying a linear curve for AY as a function of the amount of α-amylase added during the cooking process (Figure 4). This means that when the concentration of α-amylase increases, more alcohol is produced. This was to be expected given that α-amylase is required for successful liquefaction, and the higher the α-amylase activity, the more successful the starch-to-sugar conversion will be. 

The interaction plots (Figure 5) depict some of the most significant observations from the research. They show how the interaction of two independent factors can affect the dependent variables at each of the three levels considered. The least significant element, time, was investigated first. The findings show that time has a minor impact on AY, with little variation in AY across each of the timeframes tested. Temperature had the largest impact on the system and so is considered the most important variable. On preliminary inspection of time versus temperature, it is clear that AY yields vary dramatically, with 72 °C and 82 °C achieving over 360 LA/tonne throughout all time periods, while 92 °C achieved less than 360 LA/tonne (Figure 5). This means that the ideal temperature for AY is between 72 °C and 82 °C. It is also worth noting that as the duration lengthens, AY across these temperature profiles became equal. These findings can be attributed to processability issues. Water evaporation and starch swelling occurred at a faster rate when samples were cooked at 92 °C, indicating the need for extra liquor to ensure successful liquefaction of starch to sugars, which is a possible reason for lower yields. Furthermore, it is known that enzymes work best in a thicker mash; yet, if only a small amount of liquor is present, higher temperatures may result, thus inactivating the enzymes. Additionally, while the α-amylase used was a high-temperature-tolerant enzyme, the optimum temperature for the enzymes chosen was 80 °C. This impact can be seen in the graph (Figure 5), which compares temperature and α-amylase. Temperature against α-amylase exhibits polynomial curves, with a maximum AY shown. The best temperature for effective liquefaction with the α-amylase employed was 75 °C to 80 °C, which corresponds to the optimum temperature at which this enzyme impacts AY. 

The interaction plots also show the impact that α-amylase has on the process. α-amylase is the only enzyme used to convert starch to fermentable sugars during cooking. When the temperature is at ideal conditions, the addition of α-amylase increases AY, with 66 U/g producing the maximum yields of over 440 LA/tonne. At 72 °C, both 0 and 33 U/g α-amylase produce identical AY, but 66 U/g produces 10 LA/tonne more. As the temperature profile varies, the variances in AY across the different α-amylase range shifts, with larger deviations being recorded (Figure 5). Amounts of 66 U/g α-amylase consistently outperform the other factor levels across all temperature profiles. This should be regarded as the ideal α-amylase concentration to use in the lab-scale protocol of analysing AY from wheat grains. Despite earlier research demonstrating the relevance of Ca ions for stabilising α-amylase, analysis of variance showed no two-way interaction (Section 3.2.1 and Table 3) between Ca and α-amylase. When no Ca was applied, AY results were poor, with a maximum of 360 LA/tonne at each dose rate of α-amylase. When 100–200 mg/L Ca was introduced, AY rose to over 420 LA/tonne alcohol, with more than 50 U/g of α-amylase producing over 420 LA/tonne alcohol. It can be shown that Ca and α-amylase do interact with one another in order to affect AY. The AY obtained at 66 U/g α-amylase, for example, shows a significant difference (*p*-value 0.021). 

Overall, the functional relationship reveals a great deal about process optimisation, namely how the independent factors interact to influence the dependent variable. This paired with statistical analysis elucidates the role of each variable and its significance in the process. Temperature is one of the most important governing variables. In order to determine the optimum cooking process parameters, these data must be compared to other experiments, and the overall yields must be considered.

#### 3.2.3. Overall Alcohol Yield

Reviewing the overall AY achieved is one of the RSM model’s final processes in order to determine the best process parameters. Various AY yields were achieved during the study, ranging from 240 LA/tonne to over 400 LA/tonne in some cases (Figure 2). Reviewing plots of AY LA/tonne for each parameter as well as the regression equation established during this investigation can be used to optimise response.

**Figure 6:**
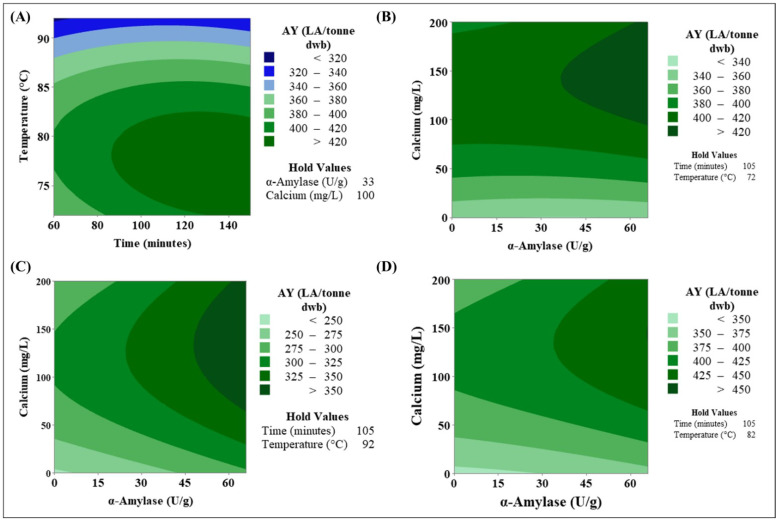
Contour plots examine the effect independent variables have on the overall AY achieved during RSM: (**A**) The effects of time and temperature on AY, with (**B**–**D**) examining the combined effects of α-amylase (U/g) and calcium (mg/L) over a time of 105 min with different temperature profiles, as indicated in the hold values.

Following a review of the data collected during this study, three optimal parameters were identified (temperature, α-amylase, and Ca) (Table 5). Over 430 LA/tonne dwb was reached in each set. Both of these options resulted in high alcohol levels. Two of the options were performed at 82 °C for the same amount of time and concentration of Ca but with different levels of α-amylase and achieved AY greater than 450 LA/tonne dwb. A difference of 4 LA/tonne dwb was observed between samples using 33 and 66 U/g α-amylase, which is an insignificant difference. Processability issues were observed during cooking with samples containing more α-amylase (66 U/g), liquefying the gelatinisation starch at a faster pace than samples containing 33 U/g α-amylase and thus indicating that more effective starch-to-sugar conversion was occurring. Following this, the other ideal cooking settings were 72 °C for a slightly longer time period (150 min) with 66 U/g-amylase and 200 mg/L Ca. Yields were slightly lower than at 82 °C but still exceeded 430 LA/tonne. Even though this option produced less alcohol, the possibility for cost savings measures while also potentially decreasing carbon footprint by performing a cooking process at such low temperatures must be explored. After evaluating the data further, it was obvious that a cooking temperature of 92 °C produced the lowest alcohol levels, ranging from 230 to 331 LA/tonne dwb depending on the other process parameters (Figure 6A). According to contour plots, the best AY was obtained when the cooking temperature was less than 80 °C, and the flour was cooked for more than 90 min. It is also worth noting that, as the cooking temperature drops, a longer cooking time frame may become necessary. This suggests that lower cooking temperatures are more effective at increasing AY.

**Figure 7:**
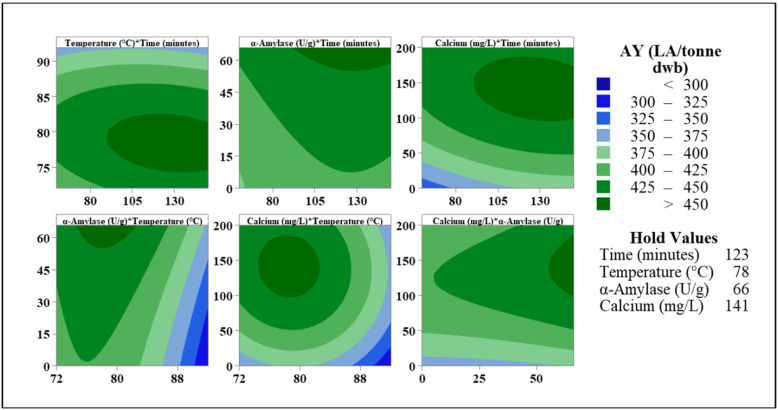
Contour plots displaying the mean AY achieved for each set of parameters when the independent variables are set at the optimal levels to achieve the highest AY.

Previous researchers achieved AY ranging from 425 to 482 LA/tonne [3,4,9], which is similar to the results obtained in this investigation. These levels can also be attained when the cooking temperature is reduced further, according to the findings of this study (Figure 7). However, one of the most significant differences in this method compared to other research is the incorporation of α-amylase and amyloglucosidase in addition to the malt inclusion rate. Furthermore, the focus of this research was on AY from cv. Costello, a hard wheat, which is often cultivated in the Irish tillage sector. Distillers dislike hard wheat because of perceived processability difficulties, such as increased foaming during fermentation, increased viscosity, and extra water adsorption during cooking. It is hoped that by adding enzymes, these difficulties associated with hard wheat can be alleviated. The effects of temperature on cooking are a significant result in this study; it acts as a regulating step and thus requires additional investigation. The effects of AY cooking temperature on different wheat endosperm kinds and cultivars will be discussed in Section 3.3.

Calcium and α-amylase do not interact, but they can have a substantial impact on the AY obtained, as indicated when the top three optimal levels were evaluated (Figure 6A–C). When the time is set to 105 min, and the temperature profile varied between levels in the study design, contour plots (Figure 6A–C) show AY through the interaction of Ca and α-amylase. The best AY is produced when calcium levels are more than 100 mg/L at all three temperatures. The maximum AY (>350 LA/tonne dwb) is reached at 92 °C when more than 50 U/g α-amylase is present during cooking. When utilizing >40 U/g α-amylase at 72 °C and 82 °C, over 420 LA/tonne dwb can be obtained. This revealed that less α-amylase is required to liquefy the starch to sugars at lower temperatures. The optimum temperature for α-amylase, which was used in this study, is 80 °C, which could explain why high AY levels are reported at these temperatures due to optimum enzyme activity. Furthermore, it was observed that when the optimum ratio of amylase to Ca ions is achieved, higher AY is achieved; when no Ca is introduced, AY is less than 350 LA/tonne dwb at all temperatures. In order to confirm this, studies investigating starch hydrolysis will be required to establish how they interact and to determine ideal ratios.

Contour, factorial, and interactions plots are useful tools for determining the optimal parameter for achieving the maximum AY, but they have limits [16]. The key challenge is the division of AY into groups; it is evident which process parameters are the most efficient, but regression analysis is required to establish the most optimum parameter. During the analysis of RSM, the regression equation (RE) was determined, taking into consideration both linear, quadratic, and interaction factors. The following equation was formed for AY:

(3) $$\begin{array}{cc} AY\;\left(LA.Tonne\right) & =-2782+3.32A+76.4B-3.08C+1.604D-0.00573A^{2}\\ & -0.483B^{2}+0.0032C^{2}-0.0044D^{2}-0.196AB\\ & =0.0045AC-0.00195AD+0.00388BC-0.00195BD\\ & +0.00227CD \end{array}$$

The ideal parameter for each of the four independent variables can be obtained using this equation. The optimum AY that may be attained within this study was 458 LA/tonne dwb, based on the regression equation and response optimisation. Using 141 mg/L Ca, 66 U/g α-amylase, and a temperature profile of 78 °C for at least 123 min, this AY was accomplished. Figure 7 shows a contour chart for these optimum settings. When reviewing the variable temperature, it was observed that temperatures between 75 and 85 °C resulted in the highest AY (Figure 6). This is supported by the graph, which compares the effects of temperature on time, calcium, and α-amylase. The ideal parameters for α-amylase have the shortest windows, with >450 LA/tonne observed when Ca was between 110 and 150 mg/L Ca, when cooking duration was 105 min, and when the temperature was within range. All of this suggested that the optimum cooking parameters had been obtained. The key variations between this procedure and that utilised in Scotland are the inclusion of enzymes and the type of wheat used. The results obtained in this study using hard wheat appear to be similar to those obtained with soft wheat samples previously published [9,10,11]. It was also shown that a lower temperature is more beneficial in achieving higher AY. When comparing the ideal parameter while adjusting the cooking temperature, there is a significant difference in AY (Figure 6 and Figure 7). The AY was 437 LA/tonne when the cooking temperature was 72 °C, a reduction of over 20 LA/tonne (Figure 7) when compared to AY at 78 °C. When the cooking temperature is 92 °C, an AY of 375 LA/tonne dwb was obtained, and the biggest difference was observed. This is a reduction of more than 82 LA/tonne, implying that this temperature should be carefully considered before being used. A difference of 6 LA/tonne dwb was observed between 82 °C and 78 °C, with the latter resulting in more alcohol being produced. This is the temperature profile that is closest to the ideal, but it is still lower than what is currently reported in the literature [9,10,11,17,18]. Since this optimal process was created with hard wheat in mind, it is now critical to investigate the AY achieved using various temperature profiles and wheat varieties. Section 3.3 will consider this factor.

#### 3.2.4. Predictive Analysis

The final component of the RSM model was to investigate the accuracy of the regression equation to see if it could be applied to predict AY. A random parameter was chosen, which is listed in Table 2, to determine the accuracy of the RE (Equation (3)). The parameter was tested in triplicate, and the average experimental AY (AYexp) was calculated. The predictive AY (AYcal) can be calculated using the regression equation. The mean percentage error was evaluated in order to determine if the model was under or overperforming. In general, the model underperformed in each example. This was to be expected. User error, environmental problems, and minor processability concerns can all contribute to poor performance. The third run, which employed a temperature of 92 °C, had the highest MPE. As previously stated, the greater temperature caused minor processability concerns in that surplus water was absorbed/evaporated, making liquefaction slightly more difficult. Run four had the lowest MPE (−0.21%), with all parameters set to the ideal bar temperature profile of 82 °C. The average MPE was −2.22%, showing that the model was very well-fitted to the given run. As a result, this procedure can be considered accurate, and it can also be used in predictive analysis when determining AY using the same milling, mashing, and fermentation conditions.

Overall, the RSM model shows good fit to the data and determines the key processing parameters; however, it is important to note some limitation of the studies design. In terms of regression equation, this can only be used to predicated AY at a local level, when variables are within this study’s ranges the theoretical yield, they may not align with actual yields. This study indicates, that on average a decreases of actual AY by >2% is expected. Finally, the RSM model is assumed. The second limitation is the interaction at play; the model is useful for predicting interaction; however, further work is need to evaluate why these interaction are at play, and this has already been noted for the alpha-amylase and calcium interaction. 

### 3.3. Comparison of Soft and Hard Wheat 

**Figure 8:**
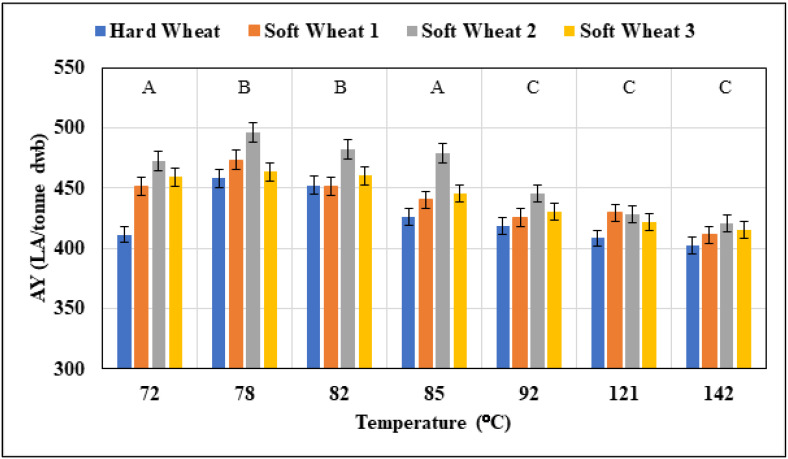
Comparison of cooking temperature for hard wheat and soft wheat samples post optimisation. All independent variables were set at optimum levels, with the temperature being the only variable altered. All results present mean ± standard deviation. Statistical analysis was employed, and samples with the same letter indicate no significant difference.

To determine the suitability of this optimum process for AY from small amounts of grain, multiple wheat varieties and endosperm types must be explored. This was accomplished by using the optimum time (12 min), calcium (141 mg/L), and α-amylase (66 U/g) parameters, as determined from the RSM model. The temperature profiles ranged from 72 °C to 141 °C. It was noted that the lower cooking temperatures achieved greater AY than that of higher cooking temperatures (Figure 8). Both high-temperature studies had AY ranging from 402.5–431.5 LA/tonne, with 121 °C outperforming 142 °C (Figure 8). This means that higher-temperature cooks are not needed to convert wheat starch to sugars for the creation of alcohol. The results observed at these temperatures are inconsistent with what has previously been published in the literature [3,4,7,9,10,17,18,19]. AY from soft wheat has been examined with a cooking temperature of 142 °C in a number of studies. Swanston et al. [19] investigated Riband, Clair, Consort, and Deban and reported yields of 450–460 LA/tonne dwb. More recently, Agu et al. [11] looked at ten different varieties of wheat and reported an AY of 446–455 LA/tonne dwb. Kindred et al. [10] also studied both hard and soft wheat and achieved yields of 431–463 LA/tonne with hard wheat (Option) and 440–467 LA/tonne with soft wheat (Riband). Additionally, Green et al. [4] investigated a soft wheat, Viscount, which is similar to soft wheat 2. Their study yielded 445–449 LA/tonne, whereas this study reports 421 LA/tonne at 142 °C and 428 LA/tonne at a 121 °C cook. This implies that the process is not performing to its full potential and that additional work may be required to optimise the mashing parameter to obtain these yields.

For all wheat varieties investigated, lower cooking temperatures performed well. Lower temperatures yielded more alcohol while also allowing for a more cost-effective and energy-efficient process. AY achieved during this study for all wheat types cooked at 85 °C was compared to AY previously reported by other researchers (Figure 8). When cooking Viscount at 85 °C, Green et al. [4] reported yields of 445–459 LA/tonne, while Costello yielded 453 LA/tonne at the same cooking temperature in this investigation, and soft wheat varieties that were evaluated all yielded over 463 LA/tonne. The primary goal of this section was to determine whether soft wheat produced more alcohol at the recommended cooking temperature of 78 °C. Hard wheat yielded 458 LA/tonne dwb when the optimum process was employed (Figure 8). Soft wheat yielded more alcohol (<10 LA/tonne depending on sample and temperature) than hard wheat, which was expected (Figure 8). Soft wheat is preferred by distillers because it is thought to contain more starch and less protein, with the starch being simpler to access since starch granules in soft wheat are loosely bound with the lipids and proteins due to increased expression of interfering grain softening proteins [20]. Due to this, it is reasonable to expect soft wheat to produce more alcohol. Soft wheat samples outperformed hard wheat samples by more than 10 LA/tonne across the board, emphasising the validity of the low cooking temperatures regardless of the wheat type employed. Soft wheat 1 yielded 473 LA/tonne dwb, about 20 LA/tonne higher than hard wheat. It was also observed that at 78 °C, it was possible to obtain 23 LA/tonne more than at 72 °C and the higher temperature of 82 °C (Figure 8). Following this, the typical cooking temperature of 85 °C produced 30 LA/tonne less than the AY obtained at 78 °C. Soft wheat 2, a benchmark distilling variety, produced the highest yields at 78 °C, yielding 485 La/tonne, a 15 LA/tonne improvement over the cooking temperature of 85 °C (Figure 8). In the past, this variety has yielded 445–459 LA/tonne when cooked at 85 °C [4]. The yield obtained at 85 °C in this investigation was 479 LA/tonne dwb, with the rise in yields likely due to the optimisation of α-amylase and Ca as well as the addition of other enzymes in mashing. Furthermore, the highest yield obtained by this sample was at 78 °C. Soft wheat 3 had the lowest yields when cooked at 78 °C but showed a substantial difference in yields when compared to the other cooking temperatures, yielding over 30 LA/tonne more than when cooked at high temperatures, while it also yielded 18 LA/tonne more at 78 °C than at 85 °C, further indicating the 78 °C is the optimal temperature for cooking. 

When the statistical analysis was performed on the samples, it was discovered that there was a significant difference in the average AY obtained (*p*-value 2 × 10^−4^) at 78 °C across the different varieties (Figure 8). Tukey post hoc analysis revealed no difference in yield between hard wheat and soft wheat 3 as well as no difference in yield between soft wheat 1 and 2, indicating that soft wheat 1 and 2 outperformed the other kinds. Following that, one-way ANOVA was utilised to see if there was any variation in the mean AY obtained across varied cooking temperatures. The null hypothesis was rejected because significant discrepancies in means were discovered. The average mean AY obtained is similar for temperature profiles 92 °C, 121 °C, and 142 °C. There was also no difference observed between 72 °C, 82 °C, and 85 °C (Figure 8). The temperature of 78 °C revealed a significant difference from all other temperatures, showing that it did achieve the maximum AY across all wheat types studied. As a result, this cooking technique may be successfully used for many varieties and endosperm types, and it yields more alcohol than other cooking temperatures. This opens the possibility of cooking at a much lower temperature than per previous reports, representing the ability to take advantage of a cost-saving and thus a more energy-efficient process. Additional research is required to determine this capability on a larger scale. Small-scale studies are suitable for testing options and determining potential, but a scale-up should be performed to establish if there is any loss in AY at increased capacity.

## 4. Conclusions

The objective of the study was to identify the most effective cooking parameters for producing the highest potential AY. In the studies, Costello hard wheat was utilised, and the RSM technique was employed to optimise the temperature, cooking period, α-amylase dose rate, and Ca concentration. The following are key findings:

- A higher AY does not require higher cooking temperatures. When temperatures of 121 °C and 142 °C were used for Costello during this investigation, AY was significantly lower compared to lower temperature (72 °C, 78 °C, 85 °C, 92 °C). The difference in AY between 142 °C and 85 °C is 25 LA/tonne, with the lower temperature providing a higher AY.
- The method was optimised using RSM, and the optimal parameters for high AY were 78 °C cooking temperature for a minimum of 123 min, with a dosing rate of 66 U/g α-amylase and 141 mg/L Ca. The alcohol yields obtained using this optimum parameter was 458 LA/tonne, which was equivalent to those published in the literature. Furthermore, the RSM model revealed that each independent variable had a unique effect on AY, with the temperature being the most important variable for better yields.
- Three soft wheat samples were studied, with different temperature profiles with ideal parameters for Ca, α-amylase, and duration, suggesting that the optimum 78 °C cooking temperature obtained the highest AY. Additionally, when soft wheat was cooked at this temperature, AY improved by more than 20 LA/tonne as compared to the cooking temperature of 85 °C currently reported in previous studies.
- This study has demonstrated that the improved technique is acceptable for both hard and soft wheat and as such reached AY comparable to, if not greater than, those previously reported by other researchers.

Further study is required to optimise saccharification (mashing) in the Irish whiskey-production process to produce a thorough approach for evaluating small batches of Irish wheat that replicates industrial production norms. Along with this, more research is needed to investigate the interaction of α-amylase and Ca during cooking, which could lead to the usage of lower dosing rates.

## Figures and Tables

**Table 1:** Independent variables employed in the RSM study at both coded and uncoded levels.

		Coded/Uncoded		
Independent Variable	Symbol	Low (−1)	Medium (0)	High (1)
Time (minutes)	A	60	105	150
Temperature (°C)	B	72	82	92
α-amylase (U/Gg)	C	0	33	66
Calcium (mg/L)	D	0	100	200

**Table 2:** Predictive analysis: experimental variables studied using the RE and AY yields achieved. The MPE and average MPE are presented. AY_exp_ is presented as mean ± standard deviation.

Sample	Time	Temperature	α-Amylase	Calcium	AYexp	AYcal	MPE (%)
	(Minutes)	(°C)	(U/g)	(mg/L)	(LA/Tonne dwb)	(LA/Tonne dwb)	
1	150	80	66	150	418.20 ± 4.55	425.60	−1.77
2	60	80	33	100	415.24 ± 2.52	425.90	−2.58
3	92	121	66	141	352.52 ± 3.59	370.62	−5.14
4	82	121	66	100	451.23 ± 1.26	452.13	−0.21
5	150	87	66	150	411.65 ± 3.40	414.91	−0.80
6	60	87	33	100	358.21 ± 6.82	368.31	−2.82
						**Average MPE (%)**	−2.22

**Table 3:** Analysis of Variance from RSM study.

Source	DF	Seq SS	Contribution	Adj SS	Adj MS	F-Value	*p*-Value	VIF
**Model**	16	98,397	89.08%	98,397.4	6149.8	6.63	0.001	
**Blocks**	2	4351	3.94%	4282.7	2141.3	2.31	0.139	1.58
**Linear**	4	53,496	48.43%	53,495.6	13,373.9	14.41	0.000	
**Time**	1	2422	2.19%	2421.9	2421.9	2.61	0.130	1
Temperature	1	30,119	27.27%	30,118.6	30,118.6	32.46	0.000	1
α-Amylase	1	6876	6.23%	6876.3	6876.3	7.41	0.017	1
Calcium	1	14,079	12.75%	14,078.8	14,078.8	15.17	0.002	1
**Square**	4	34,174	30.94%	34,173.9	8543.5	9.21	0.001	
Time*Time	1	17,893	16.20%	341.2	341.2	0.37	0.555	2.84
Temperature*Temperature	1	12,075	10.93%	5924.6	5924.6	6.38	0.025	2.84
α-Amylase*α-Amylase	1	144	0.13%	31.4	31.4	0.03	0.857	2.84
Calcium*Calcium	1	4061	3.68%	4061.3	4061.3	4.38	0.057	2.84
**Two-Way Interaction**	6	6377	5.77%	6376.6	1062.8	1.15	0.391	
Time*Temperature	1	1239	1.12%	1239.3	1239.3	1.34	0.269	1
Time*α-Amylase	1	7	0.01%	7.0	7.0	0.01	0.932	1
Time*Calcium	1	1237	1.12%	1236.9	1236.9	1.33	0.269	1
Temperature*α-Amylase	1	2623	2.37%	2623.4	2623.4	2.83	0.117	1
Temperature*Calcium	1	370	0.33%	369.9	369.9	0.40	0.539	1
α-Amylase*Calcium	1	900	0.81%	900.0	900.0	0.97	0.343	1
**Error**	13	12,063	10.92%	12,063.4	928.0			
**Lack-of-Fit**	10	11,297	10.23%	11,297.0	1129.7	4.42	0.124	
**Pure Error**	3	766	0.69%	766.4	255.5			
**Total**	29	110,461	100.00%					

**Table 4:** RSM analysis: model summary.

S	R-sq	R-sq (adj)	PRESS	R-sq (pred)	AICc	BIC
30.4623	89.08%	75.64%	69,361.8	37.21%	363.22	326.26

**Table 5:** The optimum AY achieved during the RSM run. The table displays the top three parameter setups that achieved the highest AY during experiments.

Sample	Time	Temperature	α-Amylase	Calcium	AY
	(Minutes)	(°C)	(U/g)	(mg/L)	(LA/Tonne dwb)
1	105	82	33	100	454.72 ± 1.25
2	105	82	66	100	450.25 ± 2.52
3	150	72	66	200	432.85 ± 2.69

## Data Availability

The datasets generated for this study are available on request to the corresponding author.
